# High-flow nasal cannula oxygen therapy versus conventional oxygen therapy in patients after planned extubation

**DOI:** 10.1186/s13054-019-2606-3

**Published:** 2019-11-05

**Authors:** Meng-Si Luo, Guan-Jiang Huang, Lun Wu

**Affiliations:** 1 Department of Anesthesiology, Zhongshan Hospital of Traditional Chinese Medicine, Affiliated to Guangzhou University of Chinese Medicine, 3 Kangxin Road, Zhongshan, 528400 Guangdong China; 2 Department of Otorhinolaryngology, The Second Affiliated Hospital, School of Medicine, Zhejiang University, 88 Jiefang Road, Hangzhou, 310009 Zhejiang China

We read with great interest the recent systematic review and meta-analysis of high-flow nasal cannula (HFNC) oxygen therapy versus conventional oxygen therapy (COT) in patients after planned extubation [1]. We greatly appreciate Zhu Y and colleagues’ efforts, but some important issues may better be discussed.

First, randomized controlled trials (RCTs) and non-RCTs may be inappropriately combined together in the meta-analysis, which goes against the principle of pooling studies with the similar design [2, 3]. Thus, results from RCTs and non-RCTs may better be separately pooled (Fig. 1a, b). Our Fig. 1 a and b show that the pooled results of RCTs and non-RCTs were not entirely consistent and subgroup analyses significantly decreased the heterogeneity, which suggested that the heterogeneity may originate from pooling studies with the different design. While we found that the pooled results of RCTs were even more biased in favor of HFNC than non-RCTs.

**Fig. 1:**
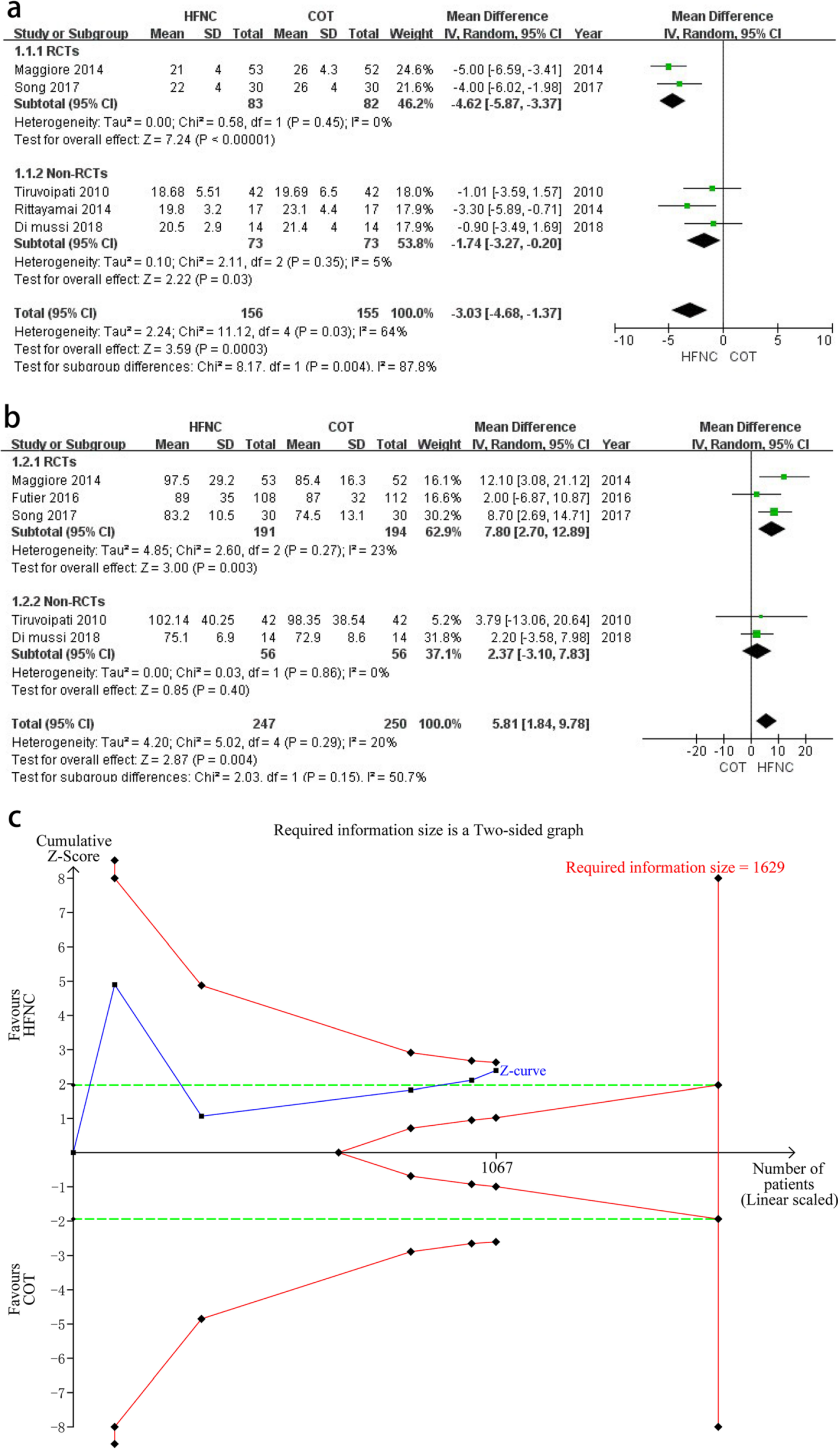
**a** Comparison of respiratory rates between the HFNC group and COT group. **b** Comparison of PaO_2_ between the HFNC group and COT group. **c** Trial sequential analysis for comparison of postextubation respiratory failure between the two groups

Second, using the standardized mean difference as the summary statistic for the meta-analyses of PaO_2_ and respiratory rates may be improper. The standardized mean difference is utilized as the summary statistic in the meta-analysis when the trials all assess the same outcome, but measure it in various ways [3]. Moreover, the standardized mean difference is unitless, which only shows the difference in a relatively measurement scale rather than a real difference in variability [3]. Therefore, the mean difference may better be used as the summary statistic to pool data (Fig. 1a, b).

Third, trial sequential analysis for comparison of postextubation respiratory failure between two groups may better be drawn based on the accurate relative risk reduction (RRR) of 37.17% ($\frac{\frac{118}{534}-\frac{74}{533}}{\frac{118}{534}}$). Then, Fig. 1c is drawn to show that the line of cumulative *Z*-curve neither crossed the line of the trial sequential monitoring boundary for benefit nor the required information size boundary, which established inconclusive evidence [4]. But in the authors’ Figure S7, the line of cumulative *Z*-curve obviously crossed the line of the trial sequential monitoring boundary for benefit, which may mislead the interpretation because of the inaccurate trial sequential analysis. Therefore, the figure of trial sequential analysis may better be not drawn based on a rough estimated RRR.

## Data Availability

Not applicable.

## Abbreviations

HFNC: High-flow nasal cannula
COT: Conventional oxygen therapy
RCT: Randomized clinical trial
RRR: The relative risk reduction
