# Supplementary material for: High-flow nasal cannula oxygen therapy versus conventional oxygen therapy in patients after planned extubation
Source: Crit Care. 2019 Nov 5;23:344. doi: 10.1186/s13054-019-2606-3 (PMC6829808; doi:10.1186/s13054-019-2606-3)
Supplement: Related texts

# Reply: Authors’ response

**Authors:** Youfeng Zhu, Haiyan Yin, Jianrui Wei

To the Editor,

Thank you very much for giving us the opportunity to respond to the valuable comments pointed out by Meng-Si Luo and colleagues with regard to our study [1].

First, randomized controlled trials (RCTs) and cross-over studies were not pooled together for primary outcome and main secondary outcomes in our article, only pooled together for secondary outcomes of respiratory rate, PaO_2_, and comfort score. According to the guidelines described in the *Cochrane Handbook for Systematic Reviews of Interventions*, quasi-RCTs and cross-over studies can be included for analysis, particularly when few RCTs addressing the topic of the review are identified [3]. Furthermore, for resolving the potential bias of pooling these studies together, subgroup studies with regard to study type (RCTs vs cross-over studies) were also performed in our study (Table 3 of our article).

Second, we thank Dr. Luo and agree that the weighted mean difference (WMD) should be used when outcome measurements in all trials are made on the same scale. WMD would be better for analyzing respiratory rate and PaO_2_. However, as the comfort score was measured with different scales among the included studies, the standardized mean difference (SMD) should be used.

Third, according to the user manual for trial sequential analysis (TSA), the estimation of the control group event proportion and an anticipated intervention effect are important determinants of the calculated required information size when doing TSA [5]. The anticipated intervention effect is usually expressed as a relative risk reduction (RRR). The estimation of RRR can be performed by using clinical experience and evidence from related studies [5, 6]. Furthermore, information size estimation must incorporate all sources of variation in a meta-analysis, including heterogeneity. As there was moderate heterogeneity (*χ*^2^ = 7.82, df = 4, *P* = 0.10, *I*^2^ = 49%) among the included studies, which might have been due to the heterogeneous population of patients and various treatment measures after extubation, the accurate RRR of 37.17% could not reflect the realistic RRR. Hence, basing on the RRR of 37.17% to draw the trial sequential analysis for comparison of postextubation respiratory failure between two groups is improper and may also mislead the results. Furthermore, we are cautious to our conclusion and underlined in our article that “a decisive conclusion should be made cautiously. Further large-scale, multicenter studies are needed to confirm our results.”
